# One‐year safety and efficacy results of insulin treatment simplification with IDegLira in type 2 diabetes

**DOI:** 10.1002/edm2.390

**Published:** 2022-12-03

**Authors:** Zoltán J. Taybani, Balázs Bótyik, András Gyimesi, Mónika Katkó, Tamás Várkonyi

**Affiliations:** ^1^ Department of Endocrinology Dr. Réthy Pál Member Hospital, Békés County Central Hospital Békéscsaba Hungary; ^2^ Division of Endocrinology, Department of Internal Medicine, Faculty of Medicine University of Debrecen Debrecen Hungary; ^3^ Department of Internal Medicine University of Szeged Szeged Hungary

**Keywords:** diabetes, IDegLira, overtreatment, simplification

## Abstract

**Introduction:**

This study aimed to investigate the sustained safety and efficacy of insulin treatment simplification with IDegLira in patients with type 2 diabetes and an HbA1c ≤ 7.5% (58 mmol/mol) during a 12‐month follow‐up.

**Methods:**

Seventy‐two adults with type 2 diabetes and an HbA1c ≤ 7.5% (58 mmol/mol) treated with multiple daily insulin injections (MDI) participated in the trial (age 63.8 ± 9.5 years, HbA1c 6.4 ± 0.7%, [46 ± 8 mmol/mol] body weight 92.95 ± 18.83 kg, total daily insulin dose: 43.21 ± 10.80 units; mean ± SD). Previous insulins were stopped, and once daily IDegLira was started. IDegLira was titrated by the patients to achieve a self‐measured prebreakfast plasma glucose concentration of ≥5 mmol/L to ≤6 mmol/L.

**Results:**

After 12 months, good glycaemic control was maintained, while body weight decreased significantly. Mean HbA1c changed to 6.2 ± 0.8% (44 ± 9 mmol/mol) (*p* = .109) and body weight changed by −3.89 kg to 89.06 ± 18.61 kg (*p* < .0001). The simplified treatment was safe and well‐tolerated. Percentage of patients experiencing at least one episode of hypoglycaemia was 49% during the month before simplification and 17% during the last 3 months of the follow‐up.

**Conclusions:**

Insulin treatment simplification with IDegLira in selected patients with type 2 diabetes is safe, maintains adequate glycaemic control and is associated with weight loss over 12 months.

## Graphical abstract

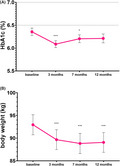

**What has this study found?**

- Simplification of insulin regimens is suitable for a lot of people with type 2 diabetes, but it is rarely carried out.
- Overtreatment is present when HbA1c is low and hypoglycaemia risk is high and when HbA1c is optimal, but the patient is using unnecessarily complex treatment instead of simpler alternatives.
- Once daily IDegLira is a potential tool for insulin treatment simplification.
- We demonstrated that switching from complex insulin regimens to IDegLira in selected overtreated patients is safe, induces weight loss and results in similar or better glycaemic control in the long term.

## 1 INTRODUCTION

It is reasonable to start insulin therapy for patients with type 2 diabetes who present with severe hyperglycaemia.^1^, ^2^ Insulin protects the beta‐cell by inducing rapid reversal of glucolipotoxicity and beta‐cell rest, and potentially contributes to the recovery of beta‐cell function.^3^, ^4^

Complex insulin regimens have potent blood glucose‐lowering effects, but are associated with hypoglycaemia and weight gain and cause significant treatment burden for the patients. As glucose toxicity resolves, the complex regimens may potentially be simplified, but due to lack of specific guidelines, deintensification is rarely carried out and many patients become overtreated.^5^, ^6^, ^7^

Patients treated with hypoglycaemic agents who have lower than optimal HbA1c values (usually HbA1c < 6.5% [48 mmol/mol]) are exposed to high hypoglycaemia risk and may be overtreated. Another form of overtreatment may be when the glycaemic status is well controlled, but the patient is using unnecessarily complex insulin regimens instead of simpler alternatives which would ensure the same efficacy with less risk of adverse events.^8^, ^9^, ^10^

Until recently, only few trials dealt with hypoglycaemic medication simplification and examined the outcomes of different deintensification regimens.^11^, ^12^, ^13^ Evidence‐based strategies for simplifying multiple daily insulin injection (MDI) treatment in overtreated people with type 2 diabetes mellitus are still lacking.

IDegLira, a once‐daily, fixed‐ratio combination (FRC) of the long‐acting basal insulin degludec and the glucagon‐like peptide‐1 receptor agonist (GLP‐1RA) liraglutide can be a tool for simplification as it provides similar glycaemic efficacy compared to basal‐bolus therapy in patients who are suboptimally controlled with basal supported oral therapy.^14^

We carried out a trial to examine the safety and efficacy of switching from MDI to IDegLira in relatively well controlled (HbA1c ≤ 7.5% [58 mmol/mol]) but potentially overtreated subjects with type 2 diabetes using low total daily insulin dose (TDD). Our preliminary 3‐month follow‐up data showed that in everyday clinical practice insulin treatment simplification with IDegLira was feasible, safe and provided similar or better glycaemic control with less hypoglycaemia and weight loss compared to the previously used complex regimens.^8^

The objective of the present paper was to assess the sustained efficacy and safety of the simplified treatment during a 12‐month follow‐up in a larger group of patients.

## 2 METHODS

This was a 12‐month, real‐world setting, prospective, one‐arm, single‐centre clinical study carried out from February 2016 to December 2019 that evaluated the safety and efficacy of switching from MDI to once daily IDegLira in selected patients with type 2 diabetes. The trial conformed to the recommendations of the Declaration of Helsinki and the International Council on Harmonization Good Clinical Practice norms with regard to medical research in humans. The protocol for this research was approved by the local institutional review board of the Békés County Central Hospital and also by the Hungarian National Medical Research Council's ethical review board. All participating patients provided signed informed consent before enrolment. The study is registered on ClinicalTrials.gov (NCT04020445).

### 2.1 Participants

Recruitment was carried out among subjects presenting on scheduled ambulatory visits for type 2 diabetes at the Diabetes Center of the Békés County Central Hospital in Békéscsaba, Hungary. Outpatients with type 2 diabetes aged ≥ 18 years were enrolled. Main inclusion criteria were as follows: random, non‐fasting serum C‐peptide level ≥1.1 ng/ml (normal range 1.1–4.1 ng/ml), HbA1c ≤ 7.5% (58 mmol/mol), MDI treatment (stable daily doses of insulin at least for 90 days prior to baseline visit [BV] ± metformin), relatively low TDD. At BV, low TDD was defined as TDD ≤70 IU/day and TDD ≤0.6 IU/kg/day at the same time. Patients reporting severe or repeated symptomatic hypoglycaemia during the month before BV using TDD ≤70 IU/day and >0.6 but <0.8 IU/kg/day could also be recruited into the study. In spite of the 70% health insurance coverage IDegLira is still a relatively costly medicine in Hungary. Only those patients who accepted the additional expenses of the treatment were enrolled.

The main exclusion criteria were type 1 diabetes, applying glucose‐lowering agents other than insulin or metformin during 90 days before BV, active cancer, anaemia (haemoglobin <100 g/L) and acute or chronic kidney disease with an estimated glomerular filtration rate < 30 ml/min/1.73 m^2^.

### 2.2 Procedures

At BV, previous insulin treatment was discontinued and once daily IDegLira was started at any time, independent of meals, repeated approximately at the same time each day. The vast majority of the patients administered IDegLira in the morning, before breakfast. The starting dose of IDegLira was 16 dosage units (each dosage unit contains 1 unit of insulin degludec and 0.036 mg of liraglutide). Patients were advised to titrate IDegLira every 3 days with 2 units to achieve a prebreakfast self‐measured blood glucose (SMBG) range of 5–6 mmol/L.^15^ The maximum daily dose of IDegLira was 50 units.

Metformin was initiated or continued and titrated up with 500 mg weekly to 3000 mg or to the maximal tolerated dose.

Patients were instructed to test blood glucose daily (at least once before breakfast and at any time when symptoms of hypoglycaemia occurred) with their own glucometer and to record their readings into their diary.

An early control (Visit 0) was performed 14 days after BV to check self‐titration and adverse events. Patients were followed during the routine diabetes care. Data were collected by the study staff at baseline and during the scheduled clinical visits performed 3, 7 and 12 months (Visits 1, 2 and 3, respectively) after BV.

### 2.3 Outcome measures

The primary endpoint was the change in HbA1c from baseline to 12 months. Secondary outcomes included change in body weight, BMI and TDD from baseline to Visit 3. The change in HbA1c was also analysed in the subgroups of patients with a baseline HbA1c ≥ 6.5% and <6.5% (48 mmol/mol). Percentage of patients experiencing at least one episode of documented (SMBG < 3.9 mmol/L) or symptomatic hypoglycaemia was assessed, and the hypoglycaemia data for the month before BV and the last 3 months of the 12‐month follow‐up were compared. Severe hypglycaemia requiring external assistance and occurrence of clinically meaningful adverse events were also recorded. Proportion of patients reaching different prespecified glycaemic targets (HbA1c < 7% and <6.5% [53 and 48 mmol/mol]) at Visit 3 were evaluated.

### 2.4 Statistical analysis

Statistical analysis was performed using GraphPad Prism 9 software (GraphPad Software). Data are presented as mean ± SD or median with interquartile range (IQR) for continuous variables in case of normal and non‐normal distribution, respectively, and as *n* (%) for frequency data. Clinical and demographic variables measured at baseline and at 3, 7 and 12 months after insulin treatment simplification were compared using repeated measures ANOVA with Fisher's LSD post hoc test for normal distributed data and Friedman test with Dunn's post hoc test for non‐normal distributed data. *p* Values < .05 were considered statistically significant.

## 3 RESULTS

Between February 2016 and December 2019, 93 MDI‐treated people were enrolled and switched to IDegLira. Soon after BV 4 persons withdrew consent (ceased therapy due to financial reasons) and 4 patients gradually reduced and finally stopped IDegLira due to repeated low SMBG values before Visit 1, and remained well‐controlled on non‐insulin treatment. Three patients discontinued IDegLira in a few days due to moderate gastrointestinal adverse effects, 1 patient had to be converted to MDI due to acute illness, 6 participants did not return to the scheduled visits, and 3 patients died during the follow‐up. Finally, 72 patients (baseline age 63.8 ± 9.5 years, HbA1c 6.4 ± 0.7% [46 ± 8 mmol/mol], BMI 33.01 ± 6.47 kg/m2, body weight 92.92 ± 18.83 kg, TDD 43.21 ± 10.80 IU/day, insulin requirement 0.48 ± 0.13 IU/kg, duration of diabetes 9.7 ± 7.5 years; mean ± SD) completed the 12‐month trial (Table 1).

**TABLE 1:** Patient characteristics at baseline and during follow‐up visits

Parameters	At baseline	At 3 months (Visit 1)	At 7 months (Visit 2)	At 12 months (Visit 3)	Estimated mean difference (95% CI) Visit 3–Baseline	p Value^a^ (Visit 3–Baseline)
HbA1c (%)	6.4 (0.7)	6.1 (0.6)	6.2 (0.7)	6.2 (0.8)	−0.2 (−0.3 to 0.0)	.109
Body weight (kg)	92.95 (18.83)	89.66 (18.69)	88.83 (18.89)	89.06 (18.61)	−3.89 (−5.35 to −2.43)	<.0001
BMI (kg/m^2^)	33.01 (6.47)	31.82 (6.32)	31.52 (6.38)	31.61 (6.22)	−1.41 (−1.92 to −0.89)	<.0001
Total daily insulin dose (units)	43.21 (10.08)	20.53 (6.49)	21.13 (7.47)	21.97 (8.16)	−21.24 (−23.41 to −19.06)	<.0001
Insulin requirement (IU/kg)	0.48 (0.13)	0.24 (0.08)	0.24 (0.09)	0.25 (0.09)	−0.22 (−0.25 to −0.20)	<.0001
Metformin dose (mg/day)^b^	1850 [0–2000]	2000 [1000–2000]	2000 [1000–2000]	2000 [1000–2000]	NA	<.001

*Note*: Values are the mean (SD) and median [IQR].

^a^ From the Friedman test followed by Dunn's post hoc test for metformin dose and from repeated measures ANOVA followed by Fisher's LSD post hoc test for other parameters.

^b^ At baseline and at 12 months visit 47 (65.3%) and 70 (97.2%) patients were taking metformin.

At baseline, 62 (86%) patients were on a basal‐bolus regimen using one dose of basal and 3 doses of prandial insulins (46 used human and 16 used analogue insulins), and 10 (14%) patients were treated with 2 or 3 doses of human or analogue premix insulins.

At BV, 47 (65.3%) patients were taking metformin (median daily dose was 1850 mg), mean number of daily insulin injections was 3.82 ± 0.54, and mean C‐peptide was 3.96 ± 2.47 ng/ml.

**FIGURE 1:**
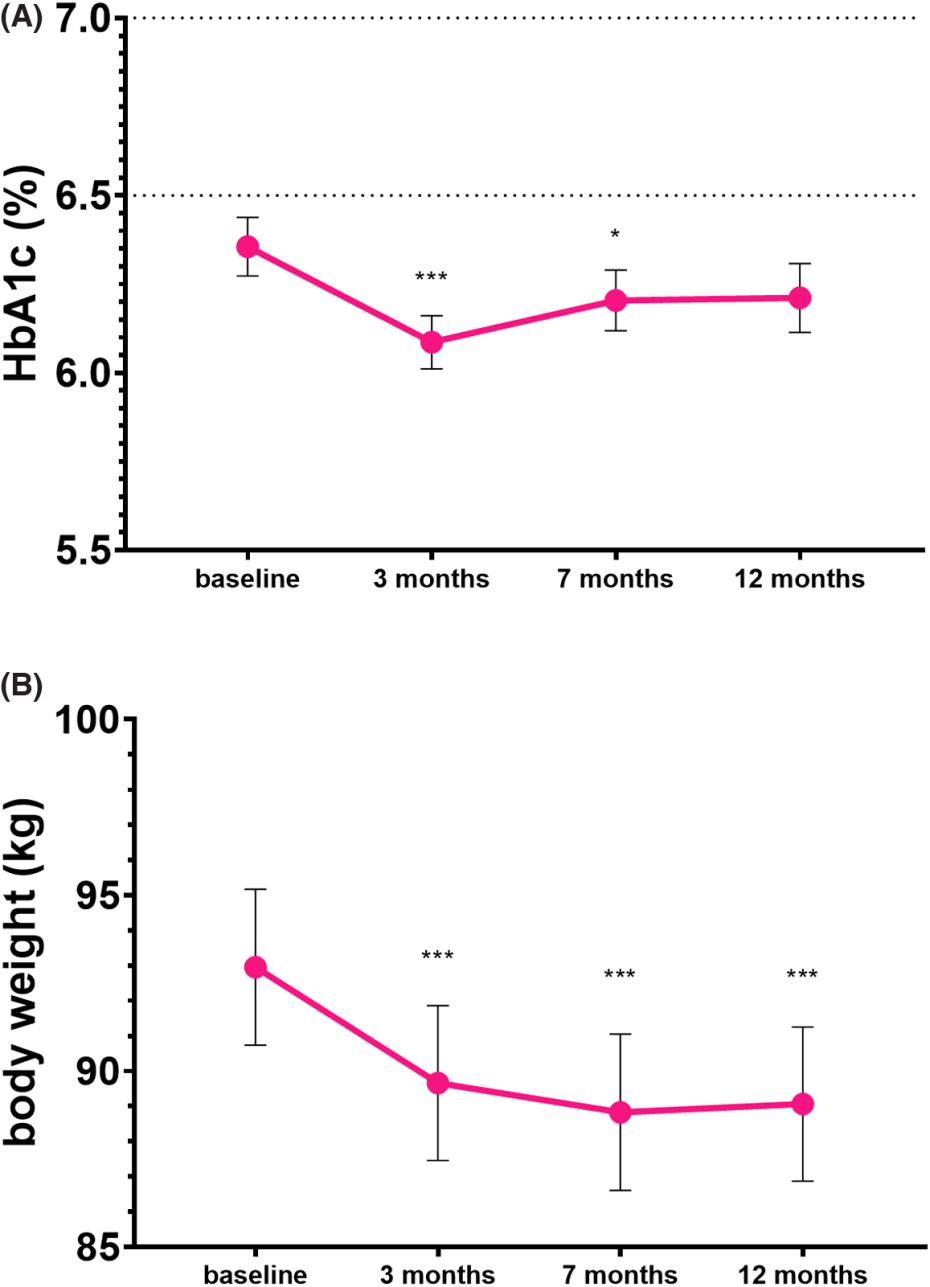
Change in HbA1c levels (A) and body weight (B) during the 12‐month follow‐up (*n* = 72). Data are presented as means ± SEM. ***p < .0001, *p < .05 compared to the baseline visit

During the 12‐month follow‐up, adequate glycaemic control was maintained by the simplified treatment (Figure 1A). Mean HbA1c changed from 6.4 ± 0.7% (46 ± 8 mmol/mol) at BV to 6.2 ± 0.8% (44 ± 9 mmol/mol) at Visit 3 (*p* = .109).

Body weight and BMI decreased significantly (Figure 1B). Body weight changed by −3.89 kg (95% CI 2.43–5.35) from 92.95 ± 18.83 kg at BV to 89.06 ± 18.61 kg at Visit 3 (*p* < .0001) and BMI changed from 33.01 ± 6.47 kg/m2 at BV to 31.61 ± 6.22 kg/m2 at Visit 3 (*p* < .0001). During the trial, 82% (*n* = 59) of the participants experienced weight loss.

Percentage of patients experiencing at least one documented or symptomatic hypoglycaemia was 48.6% (*n* = 35) during the month before simplification and 16.7% (*n* = 12) during the last 3 months of the follow‐up. Severe hypoglycaemia did not occur.

**FIGURE 2:**
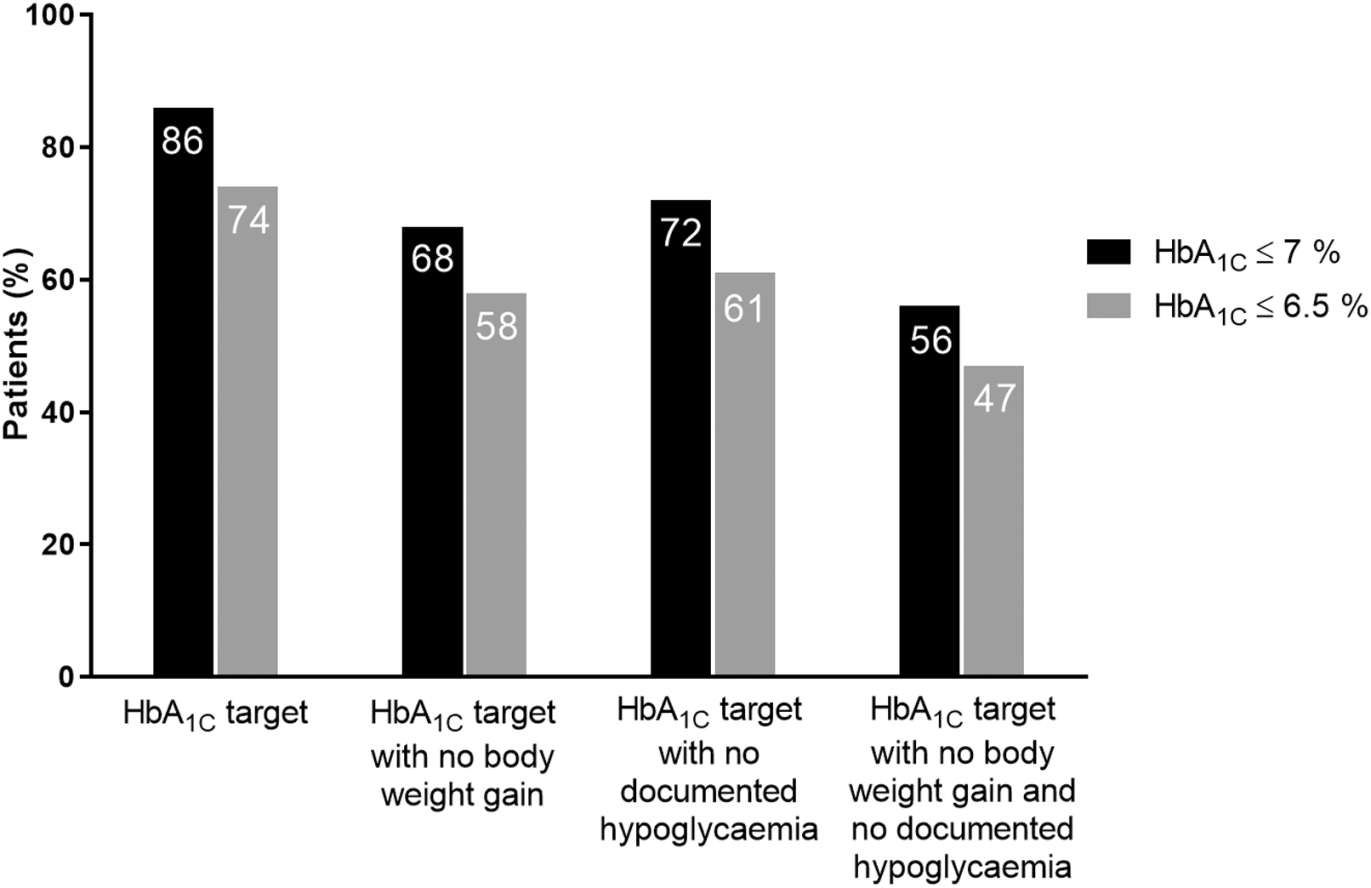
Proportions of patients who had achieved various glycaemic targets at Visit 3

Proportions of participants reaching different prespecified glycaemic targets are summarized in Figure 2.

**FIGURE 3:**
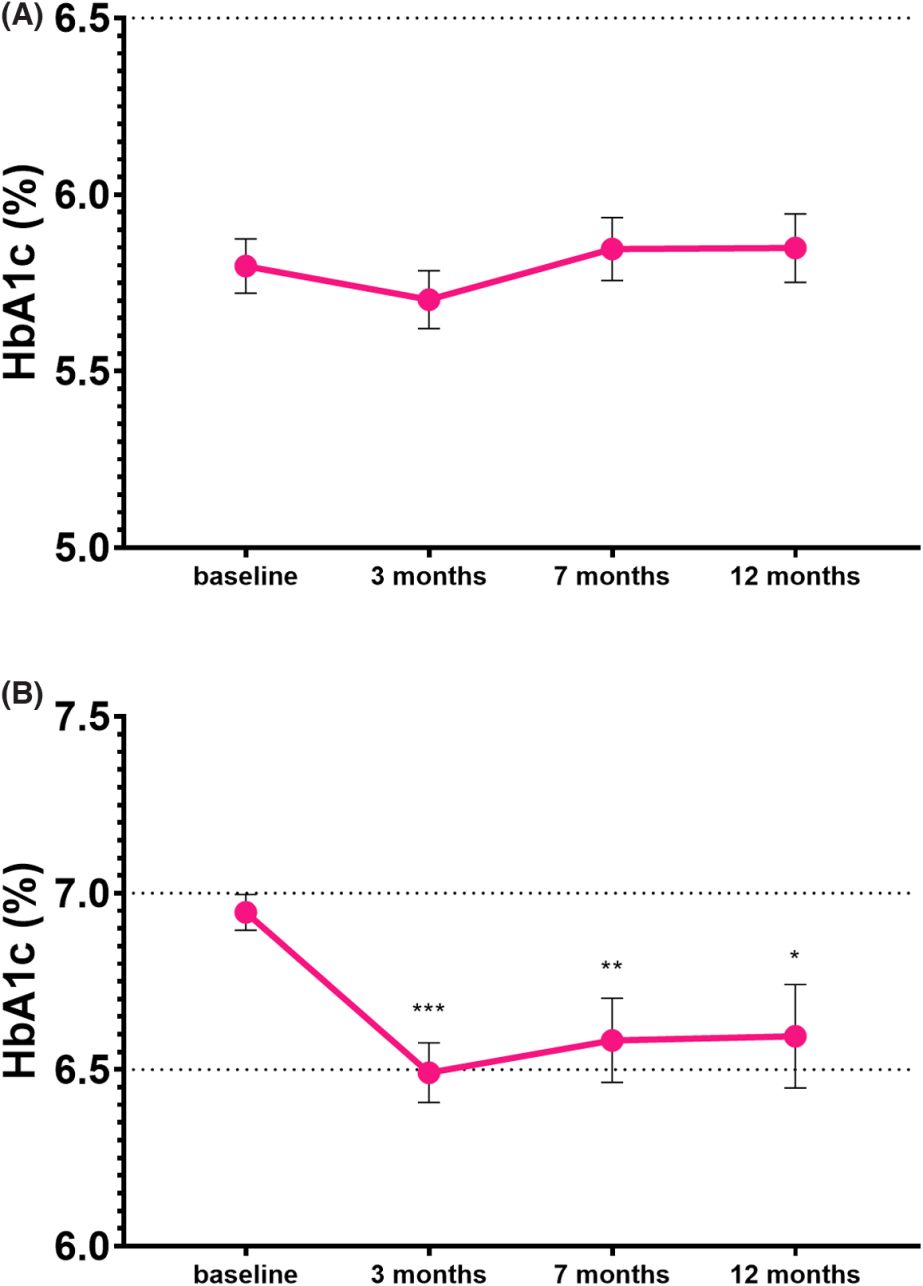
Change in HbA1c levels (means ± SEM) during the 12‐month follow‐up in the subgroup of patients (A), and with a baseline HbA1c ≥ 6.5% to ≤7.5% (*n* = 35) (B). ***p < .0001, **p < .005, *p < .05 compared to the baseline visit

In the subgroup with a baseline HbA1c < 6.5% (48 mmol/mol) (*n* = 37), mean HbA1c changed from 5.8 ± 0.5% (40 ± 5 mmol/mol) at BV to 5.9 ± 0.6% (41 ± 7 mmol/mol) (*p* = .600) at Visit 3 (Figure 3A). In this potentially overtreated group, HbA1c did not change significantly, but the risk of hypoglycaemia decreased. The percentage of patients experiencing at least one episode of hypoglycaemia was 62.2% (*n* = 23) during the month before BV and 24.3% (*n* = 9) during the last 3 months of the follow‐up.

In the subgroup with a baseline HbA1c ≥ 6.5% to ≤7.5% (48 and 58 mmol/mol) (*n* = 35), HbA1c decreased significantly (*p* = .021) from 7.0 ± 0.3% (53 ± 3 mmol/mol) at BV to 6.6 ± 0.9% (49 ± 10 mmol/mol) at Visit 3 (Figure 3B). In this group of relatively well‐controlled subjects, the simplified treatment ensured improved glycaemic status with lower hypoglycaemia risk. During the month before BV, 34.3% (*n* = 12) of the participants experienced at least one episode of hypoglycaemia while during the last 3 months of the follow‐up it was only 8.6% (*n* = 3).

After 12 months of follow‐up, the mean dose of IDegLira was 21.97 ± 8.15 dosage units (mean dose of liraglutide was 0.79 mg), 70 (97.2%) patients were taking metformin (median dose of metformin was 2000 [1000–2000] mg) and the mean insulin requirement decreased from 0.48 ± 0.13 IU/kg at BV to 0.25 ± 0.09 IU/kg at Visit 3. Mean daily number of injections changed from 3.82 ± 0.54 to 1, and the patients could also substantially reduce the daily number of blood glucose testing.

IDegLira + metformin combination was safe and generally well tolerated. Transient gastrointestinal adverse events (lack of appetite, abdominal pain, nausea, pyrosis, diarrhoea, and in 2 cases vomitus) were reported by 18 patients (25%), and 2 patients had transient dysthymia. Serious adverse events were rare. One patient had non‐fatal acute non‐ST segment elevation myocardial infarction, 1 patient was newly diagnosed with heart failure and dilated cardiomyopathy complicated with intracardiac thrombus and acute peritonitis, and 3 patients died during the follow‐up. One patient with known dilated cardiomyopathy died of acute left ventricular heart failure, while two patients were diagnosed with and died of colorectal cancer with liver metastases. In the opinion of the investigators, none of the serious adverse events and death cases were related to IDegLira + metformin combination therapy.

## 4 DISCUSSION

MDI‐treated patients who present with low HbA1c values < 7.5% and report frequent hypoglycaemia may be overtreated and similar patients without frequent hypoglycaemia may also be overtreated if they are using unnecessarily complex treatment instead of simpler options which could ensure the same efficacy with less risk and burden. It is clear that overtreated patients are among those who can benefit the most from treatment simplification. We examined prospectively the safety and efficacy of switching from MDI to once daily IDegLira in subjects with type 2 diabetes using low TDD who were considered to be overtreated (either overcontrolled or well‐controlled). Our preliminary 3‐month follow‐up data were promising but we wanted to confirm our results in a larger group of patients with a longer follow‐up.^8^

We used IDegLira for de‐escalation because beside its marked effect on fasting glucose it also has a clinically relevant impact on postprandial glucose. Moreover, the GLP‐1RA component of the drug has an insulin‐sparing effect as it enhances endogenous insulin secretion in a glucose‐dependent way. It also generates weight loss which is associated with improved insulin sensitivity and lower insulin requirement. Furthermore, the LEADER and DEVOTE trials proved the cardiovascular benefits and safety of liraglutide and degludec.^16^, ^17^ The DUAL VII trial confirmed that IDegLira has comparable glycaemic effects to MDI in patients with type 2 diabetes uncontrolled on basal insulin, but with less hypoglycaemia and a more beneficial effect on body weight. In this trial, the mean dose of IDegLira and MDI was 40 and 84 units at the end of the follow‐up. As the maximal daily dose of IDegLira is 50 units, we enrolled patients only with a TDD ≤ 70 IU/day at BV to be able to fully and securely cover the effect of the previous insulin regimen.^14^

Endogenous insulin secretion is a major criterion for the glucose‐lowering effect of liraglutide and supplements the effects of IDegLira on postprandial glucose control; therefore, we enrolled only adults who had at least partially preserved beta‐cell function. Average daily insulin production in healthy men is about 0.7–0.8 IU/kg, and the mean TDD in Caucasian men and women with type 2 diabetes treated with MDI is usually between 0.9 and 1.4 IU/kg.^14^, ^18^, ^19^ It was assumed that a normal or near normal HbA1c achieved with low TDD may refer to a partially preserved beta‐cell function. We defined low insulin need as an average TDD ≤ 70 IU/day and an insulin requirement ≤ 0.6 IU/kg/day at the same time and used these parameters together with C‐peptide to identify our potential candidates.

Our objective was to assess the sustained efficacy and safety of the simplified treatment during a 12‐month follow‐up in a larger group of overtreated patients. Our results clearly confirmed that the glycaemic control achieved with the previously used complex insulin regimes can be maintained in the longer term with IDegLira, since mean HbA1c actually remained unchanged during the follow‐up.

In the subgroup of overtreated subjects who had an HbA1c < 6.5% (48 mmol/mol) at baseline, mean HbA1c did not change significantly during the trial, but the risk of hypoglycaemia decreased markedly. In the subgroup of relatively well‐controlled patients with a baseline HbA1c ≥ 6.5% to ≤7.5% (48 and 58 mmol/mol), replacing MDI with IDegLira resulted in clinically significant decrease in mean HbA1c without increasing hypoglycaemia risk. Actually in this subgroup, the percentage of patients experiencing hypoglycaemia was substantially lower during the last 3 months of the follow‐up than during the month before BV.

Besides the beneficial glycaemic effects, insulin treatment simplification with IDegLira resulted in clinically meaningful weight loss, a reduction of insulin requirement of nearly 50% and a decrease of treatment burden. In addition, there are data which support that among older patients with type 2 diabetes taking multiple glucose‐lowering agents deprescribing with IDegLira may also improve quality of life.^20^ The observed benefits are emphasized by the fact that at Visit 3, 86.1% of our patients had an HbA1c ≤ 7% (53 mmol/mol) and 55.6% reached this goal without weight gain and hypoglycaemia.

According to our observations, IDegLira + metformin combination therapy was safe and generally well tolerated. The most frequent adverse events were gastrointestinal, transient and non‐serious, and the incidence and severity of these digestive symptoms were similar to those described in the literature.^14^ Serious adverse events were rare, and none of them was considered to be related to the antidiabetic therapy.

In our trial, we focused on overtreated patients with HbA1c ≤ 7.5% (58 mmol/mol), but insulin treatment simplification with the fixed combination of basal insulin and a GLP‐1RA may be applicable for people with suboptimal glycaemic control as well.

The post hoc analysis of the DUAL VII Japan study found that switching from low‐dose premixed insulin to IDegLira in patients with uncontrolled type 2 diabetes (*n* = 39, mean baseline HbA1c 8.26% [67 mmol/mol]) resulted in improved HbA1c and generated weight loss.^21^

The BEYOND trial demonstrated that in patients with type 2 diabetes and inadequate glycaemic control (baseline HbA1c > 7.5% (58 mmol/mol), mean HbA1c 8.6% [70 mmol/mol] at baseline) it was safe to switch from a basal‐bolus regimen to either a once‐daily FRC (IDegLira or iGlarLixi) or once‐daily gliflozin added to basal insulin, with similar glucose control, fewer insulin doses and less hypoglycaemia.^13^

Our work in line with the above‐mentioned trials draws attention to the fact that in a significant proportion of subjects with type 2 diabetes complex insulin regimens can be successfully simplified. Since clinical inertia to insulin treatment simplification has an unfavourable effect on patients, efforts should be made to avoid it.^9^

We demonstrated that simplification of basal‐bolus or premixed insulin regimens can be performed with an FRC successfully in adults with type 2 diabetes who have an HbA1c ≤ 7.5% (58 mmol/mol), are treated with relatively low insulin doses (TDD ≤ 0.6 IU/kg and TDD ≤ 70 IU/day) and have a detectable (≥1.1 ng/ml) random, non‐fasting serum C‐peptide level indicating some degree of preserved beta‐cell function. We also showed that IDegLira added to metformin to simplify treatment maintains appropriate glycaemic control at least for 12 months with less hypoglycaemia and weight loss compared to the previous insulin regimens.

Our real‐world setting, prospective, before‐after study has several limitations. It was a non‐randomized, non‐blinded, uncontrolled, one‐centred study and only Caucasian subjects were enrolled. Besides the initiation of IDegLira the initiation and/or the titration of metformin also affected the observed effects on glycaemic status, body weight and the incidence of adverse effects, but we could not estimate the strength of that effect. Our aim was to examine an IDegLira‐based strategy and not a certain medicine.

These 12‐month data confirm that in everyday clinical practice switching from low‐dose MDI to IDegLira in overtreated (well‐controlled or overcontrolled) patients with type 2 diabetes is safe, may induce weight loss, results in similar or better glycaemic control and substantially reduces insulin requirement on longer term.

## AUTHOR CONTRIBUTIONS

Z.J.T., B.B. and A.GY. designed the research. Z.J.T., B.B., M.K. and T.V. conducted the research. Z.J.T., B.B. and M.K. collected data in the electronic database. M.K. performed statistical analysis. Z.J.T., B.B., M.K. and T.V. wrote the paper. All authors commented critically and approved the final manuscript. Z.J.T is the guarantor of this work and, as such, had full access to all of the data in the study and takes responsibility for the integrity of the data and the accuracy of the data analysis.

## CONFLICT OF INTEREST

Zoltan J. Taybani received honoraria for speaking at meetings from Novo Nordisk, Sanofi, Eli Lilly, AstraZeneca, Boehringer Ingelheim and Novartis, Balázs Bótyik; received honoraria for speaking at meetings from Novo Nordisk, Sanofi, Eli Lilly, AstraZeneca, Boehringer Ingelheim and Novartis, Mónika Katkó and András Gyimesi have nothing to disclose. András Gyimesi has passed away. Tamás Várkonyi received honoraria for speaking at meetings from Novo Nordisk, Sanofi, Eli Lilly, AstraZeneca, Boehringer Ingelheim, Wörwag Pharma and Novartis. The results of the trial were presented as an oral presentation at the 14th International Conference on Diabetes Technologies & Treatments, 2–5 June, 2021, and the abstract was published in Diabetes Technology and Therapeutics, 2021, 23(S2):A54‐55, DOI: 10.1089/dia.2021.2525.abstracts. No other potential conflicts of interest relevant to this article were reported.

## Data Availability

The data that support the findings of this study are available from the corresponding author, upon reasonable request.
